# Effectiveness of Ultra-High Irradiance Blue Light-Emitting Diodes in Inactivating *Escherichia coli* O157:H7 on Dry Stainless Steel and Cast-Iron Surfaces

**DOI:** 10.3390/foods12163072

**Published:** 2023-08-16

**Authors:** Martha Minor, Luis Sabillón

**Affiliations:** 1 Department of Family & Consumer Sciences, New Mexico State University, Las Cruces, NM 88003, USA; mminor4@nmsu.edu; 2 Center of Excellence in Sustainable Food and Agricultural Systems, New Mexico State University, Las Cruces, NM 88003, USA

**Keywords:** *Escherichia coli*, low-moisture foods, blue light, dry sanitation, metals

## Abstract

The use of blue light-emitting diodes (LEDs) is emerging as a promising dry decontamination method. In the present study, LEDs emitting ultra-high irradiance (UHI) density at 405 nm (842 mW/cm^2^) and 460 nm (615 mW/cm^2^) were used to deliver high-intensity photoinactivation treatments ranging from 221 to 1107 J/cm^2^. The efficacy of these treatments to inactivate *E. coli* O157:H7 dry cells was evaluated on clean and soiled stainless steel and cast-iron surfaces. On clean metal surfaces, the 405 and 460 nm LED treatment with a 221 J/cm^2^ dose resulted in *E. coli* reductions ranging from 2.0 to 4.1 log CFU/cm^2^. Increasing the treatment energy dose to 665 J/cm^2^ caused further significant reductions (>8 log CFU/cm^2^) in the *E. coli* population. LED treatments triggered a significant production of intracellular reactive oxygen species (ROS) in *E. coli* cells, as well as a significant temperature increase on metal surfaces. In the presence of organic matter, intracellular ROS generation in *E. coli* cells dropped significantly, and treatments with higher energy doses (>700 J/cm^2^) were required to uphold antimicrobial effectiveness. The mechanism of the bactericidal effect of UHI blue LED treatments is likely to be a combination of photothermal and photochemical effects. This study showed that LEDs emitting monochromatic blue light at UHI levels may serve as a viable and time-effective method for surface decontamination in dry food processing environments.

## 1. Introduction

Shiga toxin-producing *Escherichia coli* (STEC) infection in humans is not only associated with severe gastroenteritis but also with life-threatening extraintestinal complications, such as progressive kidney failure [1]. STEC virulence attributes are linked to the production of cytotoxins known as Shiga toxins (Stx1, Stx2) or to their ability to adhere to the intestinal epithelium causing distinctive histological lesions [2]. STEC is a heterogeneous group of *E. coli* in which O157 serotypes are most commonly implicated in foodborne disease outbreaks [3].

The presence of STECs in the food supply chain continues to be a major concern for food manufacturers, consumers, and public health authorities. The Centers for Disease Control and Prevention estimates that the STEC group is responsible for at least 265,000 infections every year in the United States, of which 36% are attributed to O157 serotypes, with the remaining 64% to non-O157 STECs [4]. Epidemiological data collected from 1982 to 2002 in the U.S. suggest that animal-based products (e.g., ground beef) and raw produce are the predominant vectors of STEC O157 infections [5]. However, in recent years, *E. coli* contamination has emerged in previously unrecognized food vehicles, such as low-moisture foods (LMFs) [6,7]. Although microbial growth is inhibited by low a_w_, the long-term survival of this enteric pathogen has been demonstrated in LMFs and their processing environments [8,9]. One of the most significant risk factors for microbial contamination in dry operations is the presence of water; therefore, the use of conventional wet cleaning and sanitation procedures are restricted [10].

Outbreaks caused by LMFs not only underscore the difficulty of eradicating pathogenic bacteria from dry processing environments but also highlight the need to evaluate and incorporate innovative antimicrobial interventions. Several dry disinfection methods have been previously investigated with varying degrees of success, including ultraviolet light [11], alcohol-based sanitizers [12], gaseous ozone, and dry heat [13,14]. Over the last decade, photodynamic inactivation using visible (blue) light has emerged as an innovative and safer disinfection alternative to the aforementioned methods. Several studies have shown that blue light (405 to 470 nm) is bioactive against Gram-positive and Gram-negative pathogenic microorganisms in low moisture settings [15,16,17,18,19]. The mechanism by which blue light causes bacterial inactivation is mainly attributed to a combination of photochemical and photothermal effects. For instance, the photoexcitation of intracellular flavins and porphyrin molecules trigger the production of cytotoxic reactive oxygen species (ROS) (e.g., singlet oxygen, hydrogen peroxide) [20,21,22], which may cause irreversible cellular oxidative processes that lead to cell death [23,24]. On the other hand, radiation heat transfer from the light source to microbial cells and the surrounding environment may occur, resulting in a lethal increase in temperature during the treatment [19].

The germicidal efficiency of blue light photodynamic treatments has generally been evaluated using low irradiance levels (<100 mW/cm^2^), which requires long exposure times (several hours) to be effective [22]. Extended treatment times may preclude this technology from being deployed in high-throughput food processing environments. In addition, the effectiveness of blue light in reducing microbial contamination in dry food processing environments remains largely uninvestigated. Therefore, the objectives of this study were to (i) evaluate the effectiveness of ultra-high irradiance blue light treatments at 405 and 460 nm to inactivate *E. coli* O157:H7 sessile cells in dry stainless steel and cast-iron surfaces and (ii) examine the impact of a soil layer, using wheat flour as a model food soil system, on the antimicrobial efficacy of these treatments. The production of intracellular ROS in *E. coli* cells triggered by blue light treatments was also investigated in this study.

## 2. Materials and Methods

### 2.1. Bacterial Strains and Inoculum Preparation

Five strains of *Escherichia coli* serotype O157:H7 (ATCC: 35150, 43895, 43889, 51658, 43894) isolated from the feces of patients with hemorrhagic colitis were used in this study. Frozen stock cultures were re-activated individually by scraping the frozen broth with a sterile loop (10 μL) and transferring it into 9 mL Tryptic Soy Broth (TSB; BD Difco™ Bacto™, Sparks, MD, USA), followed by incubation at 37 °C for 22 ± 2 h. A bacterial lawn was created for each strain by spread plating 0.1 mL of overnight broth culture onto Tryptic Soy Agar plates supplemented with 0.6% (*w*/*w*) Yeast Extract (TSA-YE; CRITERION™, Hardy Diagnostics, Santa Maria, CA, USA) and incubated for 22 ± 2 h at 37 °C. The lawns were then harvested by adding 6 mL of 0.1% buffered peptone water (BPW; Difco™) to each agar plate and scraping the bacterial cells using an L-shaped spreader. Equal amounts of cell suspensions were transferred to a 50-mL sterile conical tube to create a five-strain cocktail of *E. coli* O157:H7. Bacterial cells were then harvested by centrifugation (4000× *g*/4 °C for 8 min); the resulting pellet was washed twice with 10 mL 0.1% BPW and, finally, resuspended in 4 mL of sterile distilled water.

### 2.2. Preparation and Inoculation of Metal Surfaces

Stainless steel (type 304) and cast-iron disc coupons (1.27 cm diameter, 0.38 cm depth) were used in this study. Prior to inoculation, coupons were washed thoroughly with 1% laboratory detergent solution (7X™, MP Biomedicals™, Solon, OH, USA), wrapped in aluminum foil, and subsequently sterilized by autoclaving at 121 °C for 15 min. Four sterile coupons, two of each material, were placed in sterile aluminum pans (diameter: 10 cm; Fisherbrand™, Fisher Scientific, Pittsburgh, PA, USA), and the surface spot was inoculated with 70 µL of the bacterial cocktail to achieve an approximate concentration of 9.0 log CFU/cm^2^. In addition, to evaluate the impact of organic matter on the antimicrobial effectiveness of blue light treatments, the *E. coli* cocktail was mixed with all-purpose wheat flour to emulate the presence of organic soiling at a concentration of 0.25 g/mL (0.025%). To prepare the flour slurry, 4 g of flour was placed in a 50 mL sterile conical tube and mixed with 14 mL of sterile distilled water and 2 mL of the bacterial inoculum. Seventy µL of the inoculated flour slurry was then applied to surface coupons to achieve an approximate concentration of 8.0 log CFU/cm^2^. All inoculated coupons were allowed to air-dry inside a biosafety cabinet for 3 h, with the fan running, to allow for moisture evaporation and the transition of cells from the planktonic to the sessile state.

### 2.3. The Light Emitting Diode (LED) System

**Figure 1:**
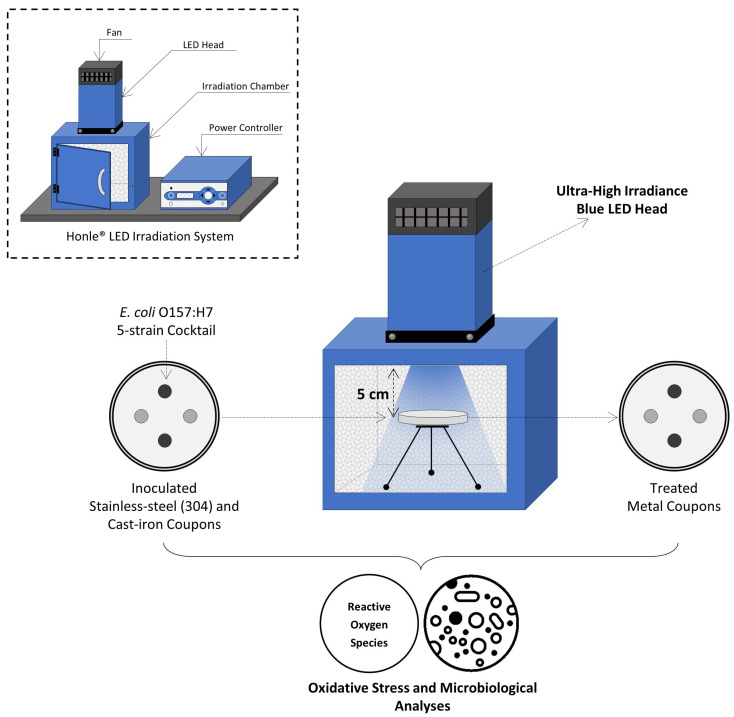
Schematic view of experimental set-up for UHI blue light treatments.

The LED system (Honle^®^ LED Cube 100 IC; Panacol-USA, Inc., Torrington, CT, USA) consisted of (i) an electronic power controller, (ii) an irradiation chamber with a reflective interior wall structure (18 × 18 × 18 cm^3^), and (iii) the LED heads emitting light at 405 nm and 460 nm wavelengths. LED lamps were mounted on top of the irradiation chamber and connected to the power controller with the LED power level set to 100%, as shown in Figure 1. Both the chamber and light heads were equipped with fans to avoid overheating during treatments. The irradiance (mW/cm^2^) of each LED head was measured using a hand-held UV meter (Honle^®^) connected to a LED spectrum surface sensor (FS LED D2; Honle^®^) positioned 5 cm from the LED head.

### 2.4. Ultra-High Irradiance (UHI) Monochromatic Blue Light Treatments

Dry, inoculated metal coupons were subjected to various ultra-high irradiance (UHI) monochromatic blue light (405 and 460 nm) treatments under equivalent energy dosages, achieved by varying the treatment times at 100% power level (Table 1). The application of equivalent energy doses allowed the comparison and determination of potential differences in antimicrobial effect between the two light wavelengths. 

The aluminum pan carrying the inoculated coupons was placed inside the irradiation chamber, leaving a 5 cm gap between the LED head and coupon surfaces. The irradiance level received by metal coupons was 842 and 615 mW/cm^2^ when using the 405 nm and 460 nm LEDs, respectively. The total energy dose delivered by a given light treatment was calculated according to Prasad and Roopesh [17] using the following equation:

*E* = *I* × *t* (1)

where *E* is the energy dose (J/cm^2^), *I* denotes the irradiance of the emitted light at a particular wavelength (W/cm^2^), and *t* represents the treatment time (seconds).

The surface temperature of the metal coupons was captured before and immediately after UHI light treatments with a thermographic camera (FLIR ONE Pro; Teledyne FLIR, Wilsonville, OR, USA), and the temperature differential (ΔT) between the two measuring points was calculated. Coupons without light treatment were used as controls. Two technical replicates (i.e., coupons) were used for each surface material and treatment, and triple-independent experiments were conducted for each treatment combination.

### 2.5. Microbiological Analysis

#### 2.5.1. Determination of Viable *E. coli* O157:H7 Cells

To enumerate the surviving *E. coli* population after UHI blue light treatments, treated coupons were aseptically transferred into 50-mL sterile conical tubes containing 10 mL of full-strength BPW and ~2 g of glass beads (2 mm diameter; BioSpec Products, Inc., Bartlesville, OK, USA). Tubes were then vortexed at high speed (3000 rpm) for 2 min to assure complete detachment of bacterial cells. This method had been previously validated in our laboratory to maximize cell recovery from coupon surfaces. The resulting bacterial suspension was then serially diluted with 0.1% BPW and appropriate dilutions enumerated by spread plating on TSA-YE plates followed by incubation at 37 °C for 24 ± 2 h. The final cell counts were expressed in Log CFU/cm^2^.

#### 2.5.2. Molecular Detection and Confirmation of *E. coli* O157:H7 Cells

For those coupons with plate counts below the limit of detection (<1 Log CFU/cm^2^), a molecular PCR-based pathogen detection system (GENE-UP^®^; bioMérieux, Inc.; Salt Lake City, UT, USA) was used to corroborate the absence of *E. coli* O157:H7. To this purpose, after serial dilution, the conical tubes containing the remaining detached bacterial suspensions in full-strength BPW were placed in an incubator at 41 °C for 24 ± 2 h. Tubes with turbid broth were pulled from the incubator, and the GENE-UP^®^ *E. coli* O157:H7 2 (ECO 2) assay was performed according to the manufacturer’s direction. In addition, the broth in these tubes was sub-cultured on Cefixime Tellurite Sorbitol MacConkey (CT-SMAC; CRITERION™, Hardy Diagnostics) agar plates and incubated at 37 °C for 24 ± 2 h for confirmation of *E. coli* O157:H7 presence based on typical colony morphology.

### 2.6. Determination of Oxidative Stress in E. coli O157:H7 Cells

The quantification of intracellular reactive oxygen species (ROS) in *E. coli* cells was performed using a general oxidative stress indicator (5(6)-carboxy-2′,7′-dichlorodihydroflourescein diacetate (Carboxy-H2DCFDA) (Invitrogen, Eugene, OR, USA), according to a procedure described by Prasad et al. [25], with minimal modifications. Briefly, LED-treated *E. coli* cells were detached from unsoiled metal coupons as previously described using phosphate-buffered saline (PBS; pH 7.4; Gibco, Life Technologies, Waltham, MA, USA). Coupons were aseptically removed and rinsed with 1 mL PBS above the 50 mL conical tube to recover the remaining bacterial cells. Cells were then harvested by centrifugation (4000× *g*/4 °C for 8 min), and the resulting pellet was washed once with 5 mL PBS. The clean pellet was then resuspended in 1 mL PBS containing 10 µM of carboxy-H2DCFDA dye and incubated in the dark at 37 °C for 30 min to facilitate the staining of cells. The stained cell suspension was centrifuged, and the resultant pellet was resuspended in 1 mL PBS. Two hundred microliters (200 µL) of the cell suspension were loaded in 0.5 mL thin-walled PCR tubes (Promega, Madison, WI, USA), and two tubes were loaded for each coupon. *E. coli* cells removed from untreated coupons were used as baseline controls, while cells treated with 200 mM hydrogen peroxide (H_2_O_2_) for 30 min at 22 °C served as positive controls. Exposure to H_2_O_2_ is widely used to induce oxidative damage/stress in microbial cells. Fluorescence values were measured using a fluorometer (Quantus; Promega Corporation, Madison, WI, USA) with excitation and emission wavelengths of 495 nm and 525 nm, respectively. The intracellular concentration of ROS was expressed as relative fluorescence intensity in arbitrary units (AU).

### 2.7. Data Analysis

Triplicate data were analyzed with statistical package software SAS version 9.4 (SAS Institute, Wake County, NC, USA), using a two- and three-way analysis of variance (ANOVA) to compare the changes in pathogen load on coupon surfaces in response to light wavelength, energy dose, and metal type. Significant differences between treatment means were determined using two- and three-way ANOVAs with Tukey’s multiple comparison test (*p* ≤ 0.05).

## 3. Results

### 3.1. Inactivation of E. coli O157:H7 on Unsoiled Metal Surfaces by UHI Blue Light Treatments

**Figure 2:**
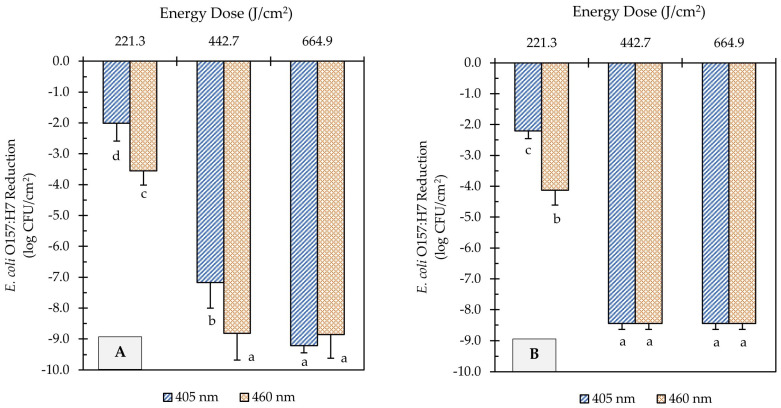
Reduction of *E. coli* O157:H7 counts in unsoiled stainless steel (**A**) and cast-iron (**B**) coupons after ultra-high irradiance blue light treatments using 405 and 460 nm LEDs. Samples were treated at a 5 cm distance from the LED head. Results are shown as mean ± standard deviation of triplicate independent experiments. Log reduction values within the same figure panel that share the same letter are not significantly different from one another (*p* > 0.05).

The *E. coli* O157:H7 sessile population on clean (no added soil) stainless steel and cast-iron coupons before UHI blue light treatments was 9.2 ± 0.2 and 8.4 ± 0.3 log CFU/cm^2^, respectively. The initial *E. coli* counts were significantly reduced by all light treatments (Figure 2). A significant effect of the treatment energy dose on *E. coli* reduction was observed for both light wavelengths (*p* < 0.05). For instance, increasing the energy dose of 405 and 460 nm light treatments from 221 to 665 J/cm^2^ further reduced the *E. coli* population by 7.2 and 5.3 log CFU/cm^2^, respectively, on stainless steel surfaces (Figure 2A). The type of wavelength also had a significant effect on *E. coli* inactivation (*p* < 0.05). At an equal energy dosage of 221 J/cm^2^, for example, the 460 nm light (3.6 log CFU/cm^2^ reduction) was more effective than the 405 nm (2.0 log CFU/cm^2^ reduction) at reducing the number of *E. coli* cells on stainless steel surfaces (Figure 2A). 

Similar inactivation trends were observed on cast-iron surfaces to those obtained on stainless steel. The *E. coli* population on cast-iron coupons was reduced from 8.4 log CFU/cm^2^ to below detectable levels after UHI blue treatments with both 405 and 460 nm LEDs at 443 or 665 J/cm^2^ energy dose (Figure 2B). Nevertheless, the molecular pathogen detection system (GENE-UP^®^) revealed that viable *E. coli* cells were still present on cast-iron surfaces. This result was later confirmed by morphological analysis of isolated colonies on CT-SMAC selective agar plates. In general, the type of metal did not have a significant effect on the reduction in *E. coli* counts (*p* > 0.05).

### 3.2. Effect of Organic Soil on the Antimicrobial Effectiveness of UHI Blue Light Treatments

LED-based treatment is a surface decontamination approach with limited penetration capacity, which might restrict its antimicrobial effect on soiled surfaces. To understand the possible shading effect of organic matter, metal coupons were soiled with a wheat flour/bacteria mixture before the application of light treatments. The mean *E. coli* O157:H7 sessile population on soiled stainless steel coupons before treatments was 8.1 ± 0.2 log CFU/cm^2^, while the population attached to soiled cast-iron coupons was 7.3 ± 0.3 log CFU/cm^2^. 

**Figure 3:**
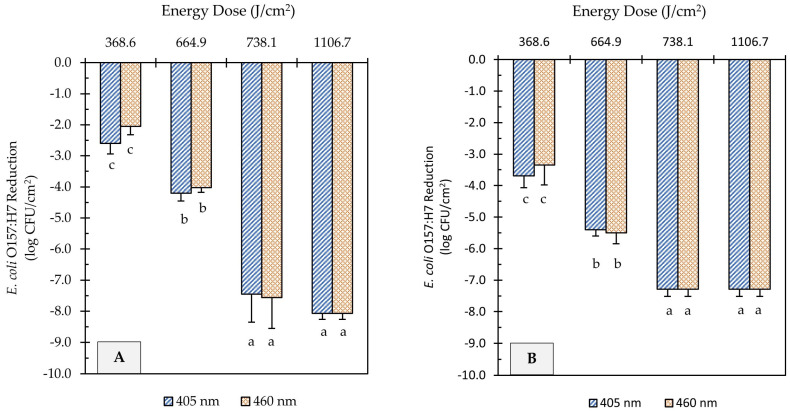
Reduction of *E. coli* O157:H7 counts in soiled (flour-coated) stainless steel (**A**) and cast-iron (**B**) coupons after ultra-high irradiance blue light treatments using 405 and 460 nm LEDs. Samples were treated at a 5 cm distance from the LED head. Results are shown as mean ± standard deviation of triplicate independent experiments. Log reduction values within the same figure panel that share the same letter are not significantly different from one another (*p* > 0.05).

UHI blue light treatments at 405 and 460 nm reduced *E. coli* O157:H7 counts on soiled metal surfaces significantly, as illustrated in Figure 3. However, higher energy doses were required to achieve comparable microbial reduction to those obtained on unsoiled metal surfaces. For example, light treatments at 665 J/cm^2^ energy dose, regardless of wavelength, were able to achieve >8.5 log reduction on clean surfaces (Figure 2), while the same treatment dose caused <5.5 log reduction on soiled surfaces (Figure 3). Increasing the energy dose to 738 J/cm^2^ further reduced *E. coli* counts by 3.3 and 1.8 log CFU/cm^2^ on flour-coated stainless steel and cast-iron surfaces, respectively. Regardless of the metal type, light treatments with the highest energy dose (1107 J/cm^2^) caused the *E. coli* population to decline to levels below the limit of detection of the plating method (Figure 3A,B). However, after incubation in enrichment broth, viable cells of *E. coli* were detected on metal coupons through a real-time PCR molecular detection system (GENE-UP^®^) and confirmed via morphological analysis of isolated colonies on CT-SMAC agar plates. Overall, the wavelength of light did not have a significant effect on *E. coli* reduction levels on soiled surfaces (*p* > 0.05); rather, log reductions were driven by the magnitude of light energy applied (*p* < 0.0001).

### 3.3. Intracellular ROS Generation in E. coli O157:H7

**Figure 4:**
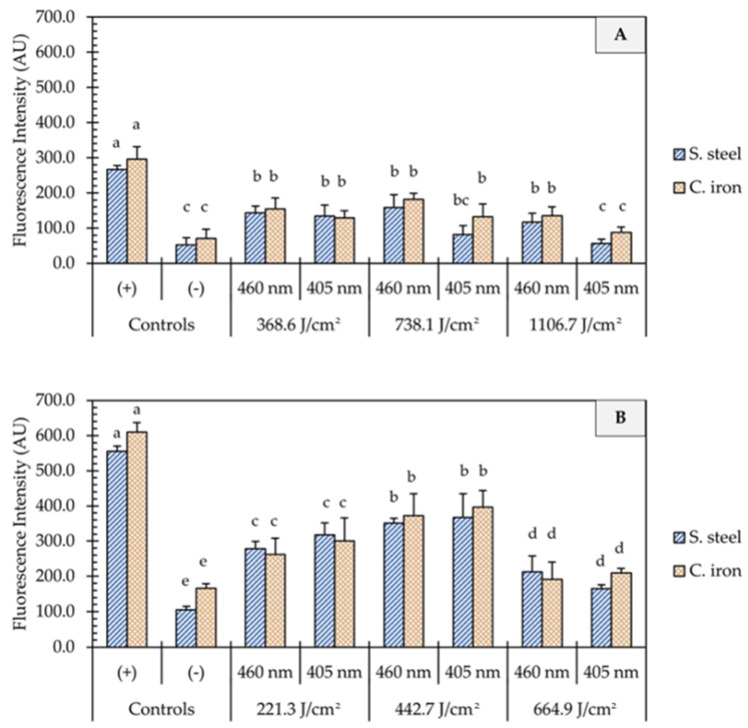
Quantification of oxidative stress in *E. coli* O157:H7 cells after the application of ultra-high irradiance blue light treatments (405 and 460 nm LEDs) on dry stainless steel and cast-iron surfaces with (**A**) and without (**B**) the presence of organic matter. Results are shown as mean ± standard deviation of triplicate independent experiments. Fluorescence intensity values within the same figure panel that share the same letter are not significantly different from one another (*p* > 0.05). Negative control (−): Untreated *E. coli* cells; Positive control (+): *E. coli* cells treated with 200 mM H_2_O_2_.

One of the proposed mechanisms of microbial inactivation using blue light is the generation of reactive oxygen species (ROS) that triggers a cascade of oxidative reactions inside microbial cells. In this study, UHI blue light treatments with 405 and 460 nm light resulted in significant intracellular ROS production in *E. coli* cells (Figure 4). In general, the generation of ROS was not influenced by the light wavelength (*p* > 0.05) but rather by the dose of energy (*p* < 0.05) and the presence of organic matter (*p* < 0.001). On clean metal surfaces, an increment in the fluorescence intensity was observed, with an increase in fluence from 221 to 443 J/cm^2^, followed by a significant decline at higher fluences of 1107 J/cm^2^ (Figure 4B). The presence of organic matter (i.e., wheat flour) on metal surfaces significantly reduced (*p* < 0.001) the generation of ROS in *E. coli* cells, as shown in Figure 4A. The average fluorescence intensity remained relatively unchanged on soiled metal surfaces, despite increases in energy dose. No significant differences in fluorescence intensity were observed between 1107 J/cm^2^ treatments and negative controls (*p* > 0.05). 

### 3.4. Temperature Changes on Metal Surfaces during UHI Blue Light Treatments

**Figure 5:**
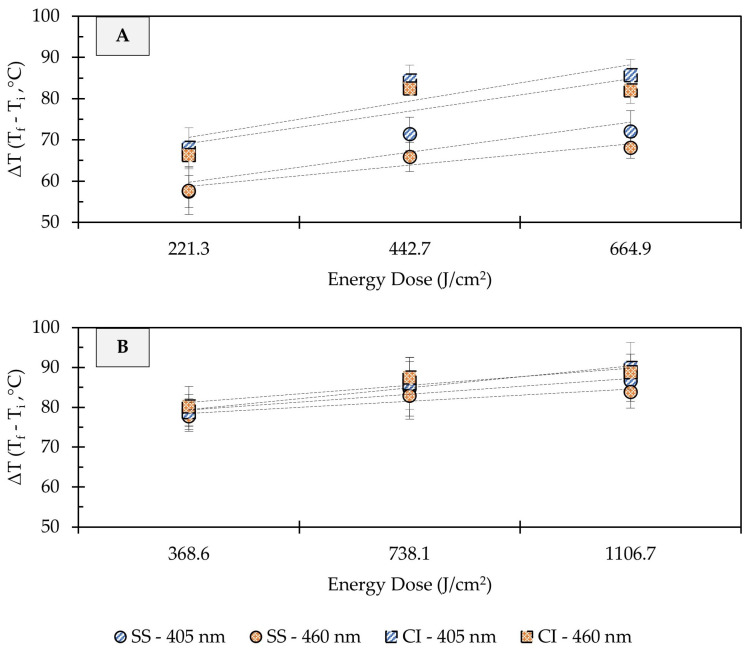
Effect of ultra-high irradiance blue light treatments on the surface temperature of unsoiled (**A**) and soiled/flour-coated (**B**) metal surfaces. Legend: SS, Stainless Steel 304; CC, Cast-Iron; Light Wavelength, 405 and 460 nm. Temperature Difference (ΔT) = Final Temperature (T_f_) − Initial Temperature (T_i_). Dotted lines denote trendlines.

Depending on treatment conditions, light-based antimicrobial interventions may increase the temperature of target surfaces, thereby inducing photothermal stress in microbial cells. In the present study, substantial increases in the surface temperature of stainless steel and cast-iron coupons were observed due to UHI treatments (Figure 5). In clean metal coupons (no soil added), the change in surface temperature (ΔT) increased linearly with the treatment energy dose, regardless of the metal type. Depending on the energy dose applied, the temperature on the surface of stainless steel metal coupons increased from room temperature (22 ± 1 °C) to 78 ± 4 or 94 ± 5 °C (ΔT: 57 ± 4 to 72 ± 5 °C) during 405 nm light treatments, while the exposure to 460 nm light caused surface temperatures to reach 79 ± 8 to 88 ± 3 °C (ΔT: 58 ± 6 to 68 ± 2 °C) (Figure 5A). Higher surface temperature increases were observed in cast-iron coupons. For example, the surface of cast-iron coupons reached 107 ± 4 and 103 ± 3 °C (ΔT: 86 ± 4 to 83 ± 3 °C) during the application of 665 J/cm^2^ energy dose with 405 or 460 nm light, respectively. In general, increasing the treatment energy dose resulted in incremental gains in temperature on metal surfaces. 

The addition of organic matter (i.e., wheat flour) to the metal coupons resulted in sharper changes in surface temperatures, with less pronounced temperature differences between treatment dose and metal types (Figure 5B). For instance, a dose of 369 J/cm^2^ with 405 or 460 nm light increased the surface temperature from 22 ± 1 to 101 ± 1 °C (ΔT: 79 ± 1 °C), regardless of the metal type. When a 1107 J/cm^2^ dose treatment was applied, the temperature of soiled stainless steel coupons reached 106 ± 5 to 109 ± 4 °C (ΔT: 84 ± 4 to 87 ± 5 °C), and 111 ± 5 to 112 ± 6 °C (ΔT: 89 ± 5 to 90 ± 6 °C) in soiled cast-iron. Previous studies have suggested that temperature increase due to LED treatments is wavelength-dependent [19]. In the present study, differences in the surface temperature of metals were observed between 405 and 460 nm treatments at the same energy dose; however, these differences were small and may not be relevant for microbial inactivation.

## 4. Discussion

In recent years, LEDs emitting blue light have emerged as a safer antimicrobial alternative to other dry sanitizing methods due to their potential to inactivate microbial cells via a combination of photochemical and photothermal effects. The results obtained in this study suggest that high-irradiance 405 and 460 nm LED treatments could serve as a viable and time-effective intervention to reduce the risk of microbial contamination in dry food processing environments. The Food Code from the U.S. Food and Drug Administration recommends that at least a 5-log microbial reduction should be achieved by an effective sanitization method [26]. In the present study, the microbial inactivation achieved by UHI blue light treatments effectively surpassed FDA-recommended levels, as >7 log reduction in *E. coli* O157:H7 counts were obtained on clean and soiled metal surfaces.

The bactericidal activity of LEDs emitting violet/blue light is primarily attributed to the intracellular generation of reactive oxygen species (ROS) due to the excitation of endogenous photosensitizing molecules (e.g., flavins and porphyrins), resulting in oxidative stress and irreversible damage to cellular components such as lipids, proteins, and enzymes [27,28]. In this study, carboxy-H2DCFDA was used as a fluorogenic probe for the detection of oxidative stress in *E. coli* cells to understand the role of ROS production in the inactivation efficacy of UHI blue light treatments. The 405 and 460 nm blue light treatments produced significant intracellular ROS generation in *E. coli* cells (Figure 4B). These results are in line with the findings of a previous study by Prasad, Gänzle, and Roopesh [25], who also reported significant ROS regeneration in dehydrated *Salmonella* Typhimurium cells when exposed to 455 nm LEDs. In the presence of organic matter, however, intracellular ROS generation in *E. coli* cells declined significantly (Figure 4A). In fact, fluorescence intensities between non-treated *E. coli* cells and those treated with 405 nm LED at 1107 J/cm^2^ did not differ significantly. This finding confirms those of a previous study by Ziuzina et al. [29], suggesting that organic matter exerts a protective effect against the antimicrobial action of reactive species generated during blue light treatments.

In addition to oxidative stress, blue LEDs have the potential to increase the temperature of target surfaces, which may have a synergistic or additive effect on microbial inactivation [30,31]. In fact, the photothermal effects of violet/blue LED (395–470 nm) treatments have not only been associated with the drying of food matrices but also with increased microbial reductions [18,19,32,33,34]. The UHI blue light treatments applied in this study led to substantial changes in the surface temperature of stainless steel and cast-iron coupons, with clear differences between both metal types. For example, clean cast-iron surfaces (ΔT: 86 ± 4 °C) registered higher temperature changes when compared to clean stainless steel (ΔT: 72 ± 5 °C) after the application of a 665 J/cm^2^ treatment with 405 nm LED (Figure 5A). These temperature differences could be due to the specific heat capacity (cp) of each metal. This thermal property refers to the amount of heat (energy) required to raise the temperature of a given type of matter [35]. The cp of many pure and alloyed metals, including cast iron (0.420 kJ/kg °C) and stainless steel (0.461 kJ/kg °C), is well established [35]. The metal coupons used in this study had the same mass (4 g each) and were exposed to equal amounts of radiant energy doses. In this context, due to its lower cp value, cast iron needed to absorb less energy to raise its temperature and to experience the largest temperature change. Slightly higher microbial inactivation rates were observed on cast-iron surfaces, which could be linked to its thermal properties (Figure 2B and Figure 3B). The increase in temperature generates an additional layer of stress on microbial cells, providing an additive effect to the generation of ROS that may enhance their inactivation rates. These findings are consistent with those of Lang, Thery, Peltier, Colliau, Adamuz, Grangeteau, Dupont, and Beney [31], who found that the thermal properties of food contact surface materials played an important role in the inactivation rates of *S. cerevisiae* using ultra-high intensity 405 nm LED treatments.

On the other hand, flour-coated coupons displayed comparable temperature changes on their surfaces, regardless of the type of metal (Figure 5B). The cp of wheat flour (1.66 kJ/kg °C) is four times higher than that of cast iron and stainless steel [36], thus requiring elevated energy doses to increase its temperature. It is also worth noting that materials with higher cp values tend to retain heat for a longer period of time [35], which may result in greater microbial reductions over time. LED illumination has limitations, such as low penetration in soiled and opaque surfaces. This limitation was evident when UHI blue light treatments were applied to flour-coated surfaces since longer exposure times (i.e., higher energy doses) were needed to increase effectiveness and achieve substantial microbial reduction levels. In general, our findings suggest that at high energy density (>665 J/cm^2^), blue light mainly produces a photothermal effect, whereas a lower energy density tends (<443 J/cm^2^) to produce both photochemical and photothermal effects. However, in the presence of organic matter, the mechanism of the bactericidal effect is more likely to be photothermal rather than photochemical. 

A variety of dry sanitation methods, such as cold plasma, dry heat, UV light, and pulsed-light technology, have been the subject of research in recent years [37]. Although promising, these technological interventions have several limitations, including low penetration levels and long treatment times, and a number of safety concerns for large-scale deployment. For instance, McKelvey and Bodnaruk [38] evaluated the effectiveness of dry heat at 80 and 100 °C against *S. enteritidis* PT30 on stainless steel carriers. The investigators observed a decline in the *Salmonella* population of 1.6 log CFU/carrier after a 4 h dry heat exposure at 80 °C, while at 100 °C, a 4.4 log reduction was noted after 2 h. More recently, Harada and Nascimento [13,14] reported reductions in *Listeria monocytogenes* (~2.2 log CFU/cm^2^) and *Bacillus cereus* (~1.8 log CFU/cm^2^) sessile cells on stainless steel coupons after a 30 min exposure to UV-C radiation at 6.5 mW/cm^2^. UV-based antimicrobial interventions, especially those with lower wavelengths (<315 nm [UV-B and UV-C]), are hazardous to end-users, as exposure is associated with skin cancer and other cell mutations [39]. The microbial inactivation levels obtained in this study were markedly higher, and achieved in a shorter period of time, than those reported in the above-cited research studies using other dry sanitation methods. Accumulating evidence indicates that, unlike conventional ultraviolet radiation, blue light is innocuous on the skin; however, prolonged exposure to blue light may damage human corneal and conjunctival epithelial cells [40]. Therefore, widespread implementation of antimicrobial blue light in food processing environments requires robust safety standards and the use of appropriate personal protective equipment, such as safety glasses. 

## 5. Conclusions

In this study, we observed that LEDs emitting 405 and 460 nm light at ultra-high irradiance (UHI) levels significantly reduced *E. coli* O157:H7 contamination on dry metal surfaces, with and without the presence of organic matter. On clean metal surfaces, the 460 nm light was more effective than the 405 nm in reducing the number of *E. coli* cells., while no differences were observed between wavelengths on soiled surfaces. Pathogenic *E. coli* counts declined by >7 log CFU/cm^2^ when 665 and 1107 J/cm^2^ treatments were applied, regardless of the light wavelength. This research demonstrates that bacterial cell inactivation induced by UHI blue light exposure is accompanied by reactive oxygen species production, as detected by the generation of fluorescence from Carboxy-H2DCFDA and an increase in temperature due to radiation heat transfer from the light source. Significant surface temperature increases were observed in UHI LED-treated coupons, which may have synergistically or additively contributed to microbial inactivation. This study also highlights the importance of removing soil from food contact surfaces before the application of light treatments since a shading/shielding effect on microbial cells may occur, thus limiting the antimicrobial effectiveness of these treatments. When applied at UHI levels, blue LEDs could overcome the need for prolonged treatment times and the use of exogenous photosensitizers, which are two major obstacles to viable implementation in the food industry. Overall, this study provided insight into the potential application of LEDs emitting monochromatic blue light at UHI levels for the control of microbial contamination in dry food processing environments. Future studies are warranted to assess the effects of UHI blue light on spore-forming microorganisms, spore germination, and other types of food contact surfaces, such as plastics.

## Figures and Tables

**Table 1:** Ultra-high irradiance treatments applied to clean and soiled stainless steel and cast-iron surfaces using 405 and 460 nm LEDs.

Organic Soiling ^a^	Light Wavelength	Treatment Intensity			
		Power Level (%)	Irradiance (mW/cm^2^) ^b^	Treatment Time (s)	Energy Dose (J/cm^2^)
None	405 nm	100	842	263	221.3
				526	442.7
				790	664.9
	460 nm	100	615	360	221.4
				720	442.7
				1080	664.1
All-Purpose Wheat Flour	405 nm	100	842	438	368.6
				790	664.9
				877	738.1
				1315	1106.7
	460 nm	100	615	600	368.9
				1080	664.1
				1200	737.9
				1800	1106.8

^a^ None: No soil added to metal surfaces; All-purpose wheat flour: Used to emulate the presence of organic soiling on metal surfaces. ^b^ Irradiance of LEDs as determined using a UV-meter/LED spectrum surface sensor at a distance of 5 cm from the light-emitting head.

## Data Availability

The data that support the findings of this study are available from the corresponding author upon reasonable request.
